# Effects of silver nanoparticles on human and rat embryonic neural stem cells

**DOI:** 10.3389/fnins.2015.00115

**Published:** 2015-04-08

**Authors:** Fang Liu, Meena Mahmood, Yang Xu, Fumiya Watanabe, Alexandru S. Biris, Deborah K. Hansen, Amy Inselman, Daniel Casciano, Tucker A. Patterson, Merle G. Paule, William Slikker, Cheng Wang

**Affiliations:** ^1^Division of Neurotoxicology, National Center for Toxicological Research/Food and Drug AdministrationJefferson, AR, USA; ^2^Center for Integrative Nanotechnology Sciences, University of Arkansas at Little RockLittle Rock, AR, USA; ^3^Division of Systems Biology, National Center for Toxicological Research/Food and Drug AdministrationJefferson, AR, USA

**Keywords:** silver nanoparticles, neural stem cells, developmental neurotoxicity, reactive oxygen species, acetyl-l-carnitine

## Abstract

Silver nano-particles (Ag-NPs) are becoming increasingly prevalent in consumer products as antibacterial agents. The increased use of Ag NP-enhanced products will almost certainly increase environmental silver levels, resulting in increased exposures and the potential for increased adverse reactions including neurotoxic effects. In the present study, embryonic neural stem cells (NSCs) from human and rat fetuses (gestational day-16) were used to determine whether Ag-NPs are capable of causing developmental neurotoxicity. The NSCs were cultured in serum free medium supplemented with appropriate growth factors. On the eighth day *in vitro* (DIV 8), the cells were exposed to Ag-NPs at concentrations of 1, 5, 10, and 20 μg/ml for 24 h. The cultured cells then were characterized by NSC markers including nestin and SOX2 and a variety of assays were utilized to determine the effects of Ag-NPs on NSC proliferation and viability and the underlying mechanisms associated with these effects. The results indicate that mitochondrial viability (MTT metabolism) was substantially attenuated and LDH release was increased significantly in a dose-dependent manner. Ag-NPs-induced neurotoxicity was further confirmed by up-regulated Bax protein expression, an increased number of TUNEL-positively stained cells, and elevated reactive oxygen species (ROS). NSC proliferation was also significantly decreased by Ag-NPs. Co-administration of acetyl-L-carnitine, an antioxidant agent, effectively blocked the adverse effects associated with Ag-NP exposure.

## Introduction

During the last decade, there has been an explosion in the development and application of engineered nanomaterials (ENM) resulting a dramatic increase in exposure to humans. While ENM can contribute positively to quality of lives by providing better materials, products, medical devices, drug delivery systems, etc., there is an urgent need to understand the potential health risks associated with exposure to such materials. A variety of toxic effects of ENM already have been shown in animal models (Elder et al., 2006; Chou et al., 2008; Poland et al., 2008; Takagi et al., 2008; Rossi et al., 2010). In particular, there is evidence from animal models that some ENM can translocate to the brain and affect its function (Oberdorster et al., 2004; Wang et al., 2007). The anti-microbial and anti-inflammatory features of silver nanoparticles (Ag-NPs) make them one of the fastest growing product categories in the nanotechnology industry. A wide variety of products, such as wound dressings, contraceptive devices, surgical instruments and bone prostheses are either coated or embedded with Ag-NPs: Ag-NPs are also incorporated into household products and textiles used in the manufacture of clothing (Chen and Schluesener, 2008). The anti-microbial properties of Ag-NPs provides the potential for their use in food packing material to increase product shelf life (Gottesman et al., 2011). Frequent exposures by multiple routes may affect human health if exposures occur above a toxicological threshold. In recent years, *in vitro* and *in vivo* studies have demonstrate that Ag-NPs are capable of causing toxicity (Braydich-Stolle et al., 2005; Hsin et al., 2008; Kim et al., 2008; AshaRani et al., 2009; Trickler et al., 2010; Mei et al., 2012). Moreover, silver as ions and NPs can cross the blood-brain barrier (BBB) and accumulate in different regions of the brain (Rungby and Danscher, 1983). Ag-NPs may be more reactive than bulk silver materials due to their higher surface area to mass ratio. These observations merit further studies on the ability of Ag-NPs to induce neurotoxicity. Zhang et al. (2013), reported behavioral dysfunction in adult rats after exposure to Ag-NPs. Compared with the adult brain, the developing brain has a rather immature BBB, whose function as a defense against exogenous insults is less effective than the mature BBB. Additionally, the proliferation and frequent turnover of cells in the developing nervous system can make it much more sensitive to environmental conditions, exogenous compounds and disease processes than the mature system. Therefore, it is important to assess the capability of Ag-NPs to cause developmental neurotoxicity which, until now, has not been explored to any significant extent. In the present study, we evaluated the ability of Ag-NPs to induce developmental neurotoxicity using both human and rodent (rat) embryonic neural stem cells (NSCs).

L-carnitine, an antioxidant dietary supplement, has been shown to attenuate brain injury associated with mitochondrial dysfunction (Zou et al., 2008; Liu et al., 2013) and has been reported to provide neuroprotective benefits in a variety of neurodegenerative and aging situations (Ishii et al., 2000; Zanelli et al., 2005; Abdul et al., 2006; Abdul and Butterfield, 2007). In the present study, acetyl-L-carnitine (an agent more efficiently incorporated into biological systems than L-carnitine) was utilized to evaluate its potential protective ability against adverse reactions associated with exposure to Ag-NPs.

Because of significant physiological, anatomical and developmental differences between the brains of humans and most animals (e.g., rodents), information obtained from most animal models are often difficult to translate to the human condition. Equally problematic, of course, is the impossibility of conducting developmental neurotoxicity studies in humans Recently, the increasing availability of stem cell-derived models, including human embryonic NSCs, with their capacity for proliferation and potential for differentiation, has provided potentially invaluable tools for examining aspects of developmental neurotoxicity including those that may be associated with exposure to Ag-NPs.

In the current study the effects of Ag-NPs were determined using cultured human and rat embryonic NSCs. The toxic effects of Ag-NPs were evaluated by testing NSC viability and proliferation after 24 h exposures to Ag-NPs: underlying mechanisms were explored as well. The results suggest that Ag-NPs significantly diminish NSC viability and that human and rat NSCs have similar vulnerability to Ag-NPs. Co-administration of acetyl-L-carnitine effectively attenuated the neurotoxicity induced by Ag-NPs. The findings from both human and rat NSCs provide relevant information on the potential health risks associated with Ag-NP exposure, which should contribute to the development of safe practices for the future use of Ag-NPs. Understanding the mechanisms by which materials such as Ag-NPs can effect toxicity should also help identify avenues of protection or prevention.

## Methods and materials

### Ag-NP preparation

Ag-NPs with an average diameter of 23 nm (which was characterized using transmission electron microscopy) were prepared as we previously reported (Mahmood et al., 2011) and were suspended in sterile water at a stock concentration of 5 mg/ml. Before each experiment, the stock solution was sonicated for 10 min in an ultrasonic water bath to ensure a uniform distribution. Handling of Ag-NP solution was carried out under a biological safety cabinet class II fume hood to keep sterile.

### NSC culture

Human NSCs were purchased from PhoenixSongs Biologicals (Branford, CT). Acetyl-L-carnitine was purchased from Sigma (St. Louis, MO, USA). Rat embryonic NSCs were harvested from embryonic/gestational day 16 Sprague-Dawley rats (the day when a mucus plug is observed is gestational day 0) bred at NCTR rodent facility. All animal procedures were approved by the National Center for Toxicological Research Institutional Animal Care and Use Committee and conducted in full accordance with the Public Health Service Policy on Humane Care and Use of Laboratory Animals (National Research Council, 2011). In brief, timed pregnant rats were euthanized by an overdose of isoflurane. After removal of the meningeal tissue, cortices from embryonic rat brains were mechanically dissociated in ice-cold Hank's calcium- and magnesium-free solution through a fire-polished Pasteur pipette and centrifuged for 10 min at 200 × g. The pellet was suspended and washed in Dulbecco's modified Eagle's medium (DMEM/F12) (Gibco by Life Technologies, Grand Island, NY, USA) by centrifuging for 10 min at 200 × g. The undifferentiated cells were evenly distributed and plated in 96-well plates, polylysine-coated coverslips in 6-well plates, or Petri dishes (10 cm), with a seeding density of 3 × 10^5^ cells/ml, in growth medium (DMEM/F12) supplemented with N-2 (Gibco by Life Technologies) and growth factors [10 ng/ml epidermal growth factor, 10 ng/ml basic fibroblast growth factor, 5 ng/ml platelet-derived growth factor, and 5 ng/ml neurotrophin-3 (Millipore, Billerica, MA, USA)]. Human NSCs were cultured under that same condition.

### Immunocytochemistry and nuclear staining

Nestin, an intermediate filament protein, is important for the proper survival and self-renewal of NSCs, and is a widely accepted marker of NSCs. SOX2 (sex determining region Y-box 2) is a transcription factor that serves as another generally employed NSC marker. The expression of nestin and SOX2 was tested in the study. A mouse monoclonal antibody to nestin (1:150, Millipore) and a rabbit polyclonal antibody to SOX2 (1:200, Abcam) were used to label rat NSCs. Briefly, the rat NSCs were rinsed with PBS before fixation with ice-cold 4% paraformaldehyde in phosphate buffered saline (PBS) and permeabilized with 0.5% bovine serum albumin (BSA)/0.1% Triton X-100 in PBS for 1 h. The cells were then incubated with primary antibodies (anti-nestin and anti-SOX2) in PBS with 0.5% BSA and 0.1% Triton X-100 at 4°C overnight. Bound antibodies were revealed with FITC-conjugated sheep anti-mouse IgG second antibody (1:80 diluted in PBS with 0.5% BSA) and Rhodamine-conjugated sheep anti-rabbit IgG second antibody (1:40 in PBS with 0.5% BSA). Cells were counterstained with 4′,6-diamidino-2-phenylindole (DAPI), a nuclear stain dye, in the mounting medium. Labeled cells were viewed using an Olympus FV1000 confocal microscope (Olympus, Center Valley, PA, USA).

Meanwhile, polyclonal rabbit anti-SOX2 (from Invitrogen, CA, USA) and polyclonal rabbit anti-nestin (from Millipore) were applied to visualize the staining of human NSCs.

### Lactate dehydrogenase (LDH) and MTT reduction assays

After the NSCs were exposed to different doses of Ag-NPs for 24 h (5 wells per dose) in growth medium, the medium was collected and assayed for LDH activity as described previously (Wang et al., 2005), using a cytotoxicity detection kit from Roche Applied Science (Indianapolis, IN, USA). NSC viability was evaluated using an MTT [3-(4,5-dimethylthiazole-2-yl)-2,5-diphenyltetrazolium bromide] assay as previously described (Wang et al., 2000).

### 5-ethynyl-2′-deoxyuridine (EdU) incorporation assay

EdU is a nucleoside analog of thymidine that is incorporated into DNA during DNA synthesis. Cell proliferation was assessed as previously described (Liu et al., 2014), using a commercially available EdU kit [Click-iT® EdU Alexa Fluor® High-throughput Imaging (HCS) Assay; Invitrogen] according to the manufacturer's instructions.

### TUNEL-assay

To assess whether Ag-NPs induced apoptosis in NSCs, a terminal deoxynucleotidyl transferase-mediated deoxy-uridine triphosphate nick end labeling (TUNEL) assay, which labels broken DNA strands that are often associated with apoptosis (Johnson et al., 1998; Wang et al., 2005) was performed.

### DCF-assay and 8-oxo-dG ELISA

Ag-NP-induced oxidative stress was evaluated by measuring the generation of 2′, 7′ –dichlorofluorescin (DCF) and 8-hydroxy-2′-deoxyguanosine (8-oxo-dG). H_2_DCFDA (Life Technologies, Carlsbad, CA) is a cell permeant used as an indicator of ROS generation in cells: the non-fluorescent H_2_DCFDA is converted to highly fluorescent DCF in an oxidative milieu. NSCs were incubated with 10 μM H_2_DCFDA in phenol red-free growth medium for 30 min at 37°C in the dark and excess H_2_DCFDA was washed out using PBS. The cells were then treated with different doses of Ag-NPs for 24 h (5 wells per dose) and fluorescence was measured using wavelengths of 492 nm for excitation and 527 nm for emission.

8-oxo-dG and its analogs were used as biomarkers of oxidative DNA damage and oxidative stress. To evaluate Ag-NP-induced oxidative stress in NSCs, an 8-oxo-dG ELISA assay was utilized as previously described (Liu et al., 2013), according to the manufacturer's instructions (Trevigen, Gaithersburg, MD, USA).

### Western blot of the pro-apoptotic gene bax

Bax expression at the protein level was evaluated using Western blotting procedures as previously described (Wang et al., 2005); Western blots of β-actin served as an internal control.

### Statistical analysis

Statistical analyses were performed and graphs were produced using GraphPad Prism5. Data are expressed as means ± SDs. Data from the LDH and MTT assays, 8-oxo-dG assay and densitometry value of Bax were analyzed using One-Way ANOVA, with Tukey's *post-hoc* analysis to compare all pairs of groups. TUNEL and EdU incorporation assays were analyzed using unpaired *t*-test. All analyses were two-tailed and considered to be statistically different when a *p* < 0.05 was determined. Each condition was assessed at least in triplicate and experiments were repeated three times independently.

## Results

On day *in vitro* (DIV) 2, the seeded NSCs were observed attaching to the bottom of culture dishes/wells. Over the following days, more bipolar NSCs emerged and began forming spheres, indicating their capability to proliferate. The experiments were performed on DIV 8.

**Figure 1:**
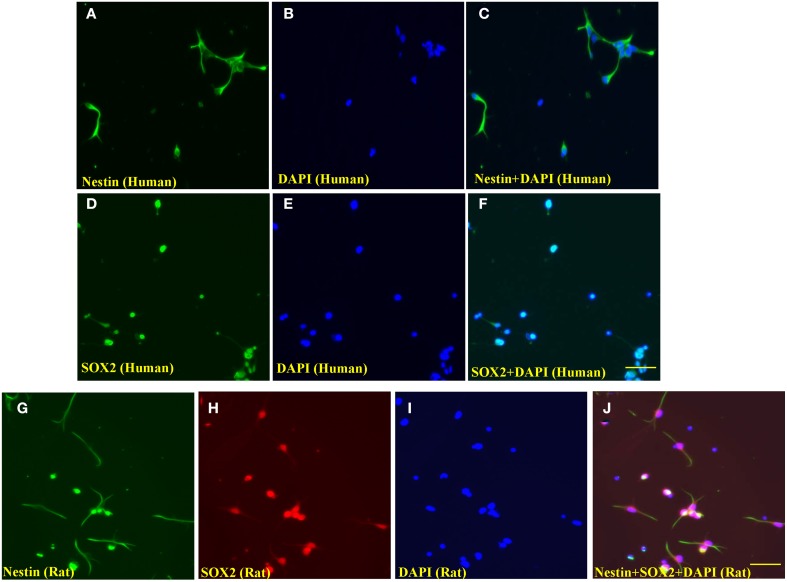
**Representative photographs of immunocytochemical staining of nestin and SOX2 of human and rat NSCs**. The evenly distributed neural stem cells (human) were positively stained with nestin (**A**, green; fluorescence) and SOX2 (**D**, Green; fluorescence), indicating these cells were undifferentiated human NSCs. The nuclei of cultured cells were stained by the nuclear dye, DAPI (**B,E** and **I**, blue). **(C,F)** are merged pictures. Similarly, rat NSCs were stained with nestin (**G**, green; fluorescence) and SOX2 (**H**, red; rhodamine), and **(J)** is a merged picture of **(G–I)**. Scale bars = 60 μm.

Both human and rat embryonic NSCs were verified using the NSC markers SOX2 and/or nestin and counterstained with DAPI. As shown in Figures 1A–F, the immunocyto-chemical stains demonstrated that the majority of the human NSCs were nestin and SOX2-positive. Similarly, most rat NSCs were stained with nestin and SOX2 (Figures 1G–J).

### Ag-NP-induced cytotoxicity in NSCs

**Figure 2:**
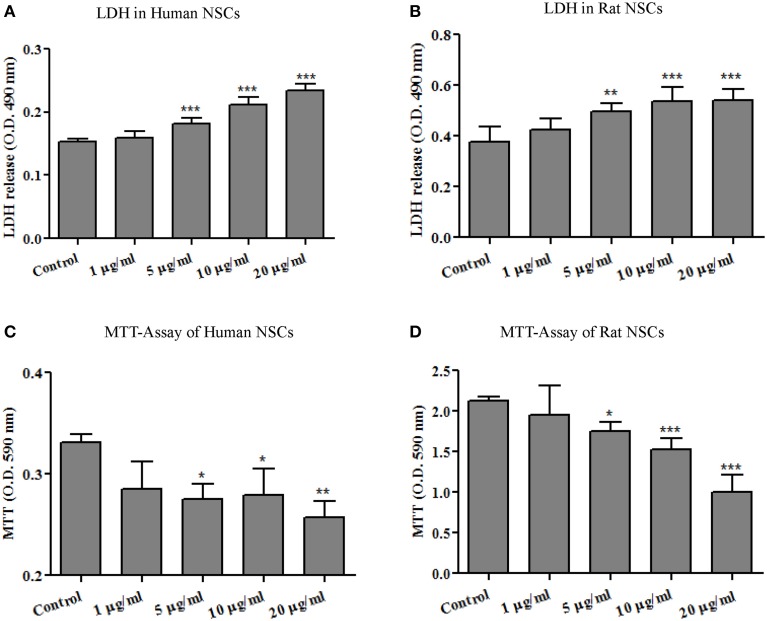
**Ag-NP-induced toxicity was evaluated by LDH release and MTT assays**. 24-h exposures of neural stem cells (**A**, human NSCs and **B**, rat NSCs) to Ag-NPs resulted in a significant dose-related increase in the release of LDH and decrease in mitochondrial metabolism of MTT into formazan (**C**, human NSCs and **D**, rat NSCs). The release of lactate dehydrogenase (LDH) into the culture medium occurs with loss of plasma membrane integrity, a process most often associated with necrotic cell death. MTT is an important indicator of cell survival. Each condition was assessed at least in triplicate and experiments were repeated three times independently. Data are presented as means ± SDs. * *P* < 0.05, ** *P* < 0.01, *** *P* < 0.001.

LDH is a cytoplasmic enzyme sequestered inside viable cells with intact plasma membranes. It is released from cells with damaged membranes, thus, the amount of LDH released from cells into the culture medium is taken to indicate some level of toxicity. As shown in Figures 2A,B, no significant effect on extracellular LDH concentration was detected at the lowest dose of 1 μg/ml. At doses of 5 μg/ml and higher, however, Ag-NP exposure resulted in significant, dose-related increase in LDH release from both human [*F*_(4, 24)_ = 69.70, *p* < 0.0001] and rat NSCs [*F*_(4, 26)_ = 13.81, *p* < 0.0001] suggesting that Ag-NPs disrupted NSC plasma membrane integrity.

The capability of mitochondria to convert MTT (3-[4,5-dimethylthiazol-2-yl]-2,5 diphenyl tetrazolium bromide) to formazan crystals is related to the number of viable cells in culture. The MTT assay is broadly used to measure cytotoxic effects *in vitro*. As shown in Figures 2C,D, MTT metabolism was decreased dose-dependently in both human [*F*_(4, 12)_ = 6.885, *p* < 0.005] and rat NSCs [*F*_(4, 25)_ = 32.21, *p* < 0.0001] after Ag-NP exposure. It should be mentioned that Ag-NP exposures lasting only 3- or 6-h did not significantly affect the viability of NSCs as evaluated by LDH release and MTT metabolism (data not shown).

**Figure 3:**
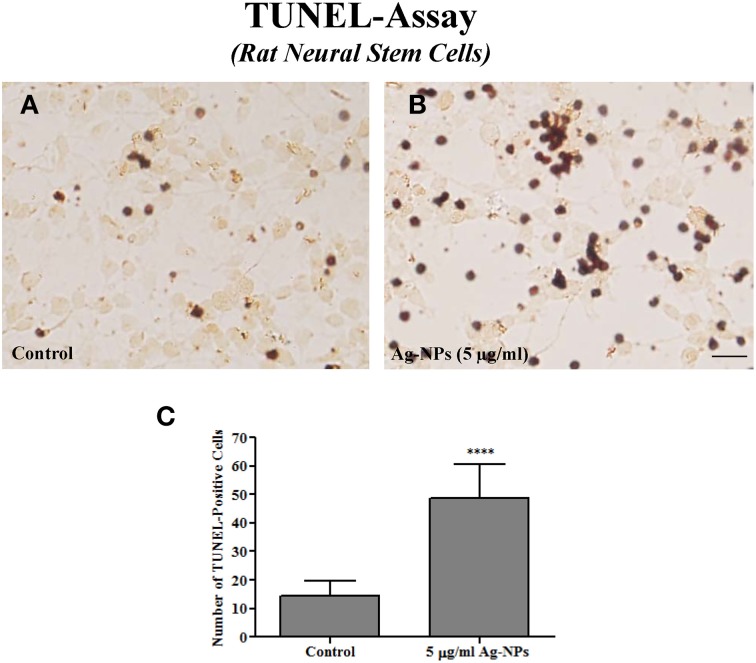
**Representative photographs of a TUNEL assay in rat NSCs**. Only a few TUNEL-positive cells were observed in the control culture **(A)**. However, numerous darkly stained TUNEL-positive cells indicating enhanced apoptosis were observed in Ag-NP-exposed culture **(B)**. Quantification [by counting the number of positively-stained cells in five 0.24 mm^2^ field (one in the center and one in each of the four corners Wang et al., 2005)] shows a significantly increased number of TUNEL-positive cells in Ag-NP-exposed cultures compared with the controls **(C)**. Data are presented as means ± SDs. **** *P* < 0.0001. Scale bar = 50 μm.

**Figure 4:**
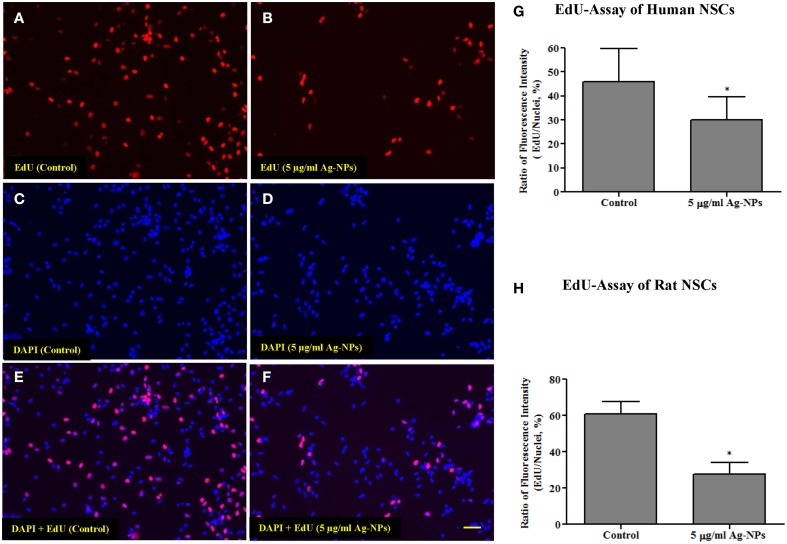
**Representative photographs from an EdU incorporation assay from human NSCs**. Both human and rat NSCs were exposed to Ag-NPs (5 μg/ml) for 24 h. The number of dividing cells was substantially decreased (**B**,**D**,**F**) in Ag-NP-exposed NSC cultures compared with the control (**A,C**,**E**). Quantitative analysis of the EdU-Alexa Fluor staining shows that the proliferation rate (ratio of the intensity of EdU-Alexa Fluor-stained nuclei to the intensity of DAPI-stained nuclei) of neural stem cells was significantly reduced in both human NSCs **(G)** and rat NSCs **(H)**, after Ag-NP exposures. Data are presented as means ± SDs. * *P* < 0.05. Scale bar = 50 μm.

Ag-NP-induced damage was also assessed on rat NSCs using the TUNEL assay. It is generally agreed that the TUNEL assay detects damaged DNA strands that are often associated with apoptotic cell death. Only a few TUNEL-positive cells were detected in control cultures (Figure 3A). However, numerous TUNEL-positive cells exhibiting typical DNA fragmentation indicative of apoptosis were observed in Ag-NP-exposed cultures (Figure 3B). 24-h Ag-NP exposures at 5 μg/ml caused a significant increase in the number of TUNEL-positive cells (control vs. 5 μg/ml Ag-NPs: 14 ± 2.00 vs. 49 ± 4.47, *p* < 0.0001, *n* = 7/group) compared with controls (Figure 4C).

### Ag-NP exposure and NSC proliferation

Proliferation is an important function of NSCs. In the present study, NSC proliferation was determined using EdU-Alexa Fluor staining. The intensities of EdU-Alexa Fluor-labeled nuclei and DAPI-labeled nuclei were determined using ImageXpress (Molecular Devices, CA, USA). Figures 4A–F show the distribution of EdU-Alexa Fluor and DAPI-stained nuclei in human NSCs. After 24-h exposures to Ag-NPs at 5 μg/ml, the number of dividing cells was substantially decreased in both human (control vs. 5 μg/ml Ag-NPs: 45.92 ± 5.27 vs. 29.91 ± 4.02, *p* < 0.05, *n* = 6/group) and rat NSCs (control vs. 5 μg/ml Ag-NPs: 60.93 ± 6.68 vs. 27.52 ± 6.56, *p* < 0.05, *n* = 3/group), as evidenced by reduced EdU-Alexa Fluor staining (Figures 4G,H, respectively).

### Elevated ROS generation and protection by acetyl-L-carnitine

**Figure 5:**
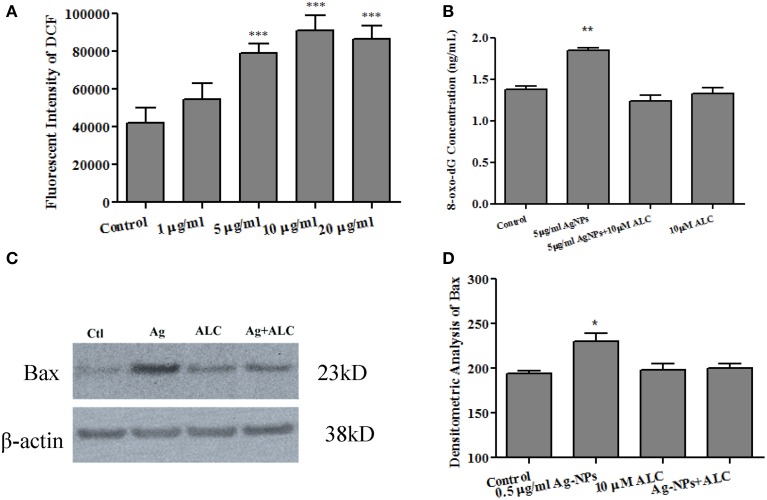
**Ag-NP-induced oxidative stress and protection by acetyl-L-carnitine**. 24 h exposure of NSCs to Ag-NPs significantly increased mitochondrial ROS generation, as indicated by elevated levels of DCF expression [**A**: *F*_(4, 13)_ = 28.03, *p* < 0.0001]. 24 h exposures to Ag-NPs also increased oxidative DNA damage as evidenced by significant increases in 8-oxo-dG expression [*F*_(3, 8)_ = 24.78, *p* < 0.001] which was effectively blocked by the co-administration of acetyl-L-carnitine (ALC) **(B)**. Additionally, the over-expression of Bax protein (a pro-apoptotic marker) induced by Ag-NPs was effectively attenuated by co-administration of acetyl-L-carnitine **(C,D)**. No significant effect was observed when acetyl-L-carnitine was administered alone **(B,D)**. Data are presented as means ± SDs. * *P* < 0.05, ** *P* < 0.01, *** *P* < 0.001.

Previous studies have indicated an ability of Ag-NPs to elevate ROS generation (Hsin et al., 2008; Rahman et al., 2009; Mei et al., 2012). In the current study, since rat and human NSCs have shown similar sensitivity in Ag-NP-induced cell death and proliferation rate, to determine whether Ag-NPs can cause an increase in ROS production, rat NSC cultures were loaded with H_2_DCFDA, which is converted to fluorescent DCF under oxidative conditions. As shown in Figure 5A, 24 h Ag-NP exposures dose-dependently increased ROS generation [*F*_(4, 13)_ = 28.03, *p* < 0.0001].

8-hydroxy-2′-deoxyguanosine (8-oxo-dG) is a modification of guanine induced by its interaction with ROS. To evaluate the involvement of ROS in Ag-NP-induced damage to NSCs, 8-oxo-dG levels in the medium were determined using an enzyme-linked immunosorbent assay (ELISA). 24 h exposures of rat NSCs to the working Ag-NP concentration of 5 μg/ml increased oxidative DNA damage as indicated by a significant elevated level of 8-oxo-dG production [Figure 5B, *F*_(3, 8)_ = 24.78, *p* < 0.001]. No significant effects were detected in cultures exposed to the same concentration of Ag-NPs for 3 or 6 h (data not shown).

To assess the ability of anti-oxidant agents to protect against Ag-NP-associated toxicity, rat NSCs were exposed to Ag-NPs at 5 μg/ml for 24 h in the presence or absence of acetyl-L-carnitine (10 μM). Acetyl-L-carnitine is a dietary supplement that has been reported to prevent apoptotic death (Mast et al., 2000; Wang et al., 2011). Although the mechanisms by which acetyl-L-carnitine exerts its protection are not clearly defined, it works as a mitochondria protector by maintaining mitochondrial homeostasis and bioenergetics (Patel et al., 2010). In the present study, acetyl-L-carnitine effectively attenuated Ag-NP-induced oxidative damage (Figure 5B). No significant effects were observed when acetyl-L-carnitine was administered alone.

The expression of the pro-apoptotic gene, Bax, was also evaluated at the protein level. Consistent with the 8-oxo-dG data, 24 h exposure of rat NSCs to 5 μg/ml of Ag-NPs caused a significant increase in Bax protein expression [Figure 5C, *F*_(3,8)_ = 6.738, *p* < 0.05]. Co-administration of acetyl-L-carnitine effectively blocked the Ag-NP-induced increase in Bax expression. When acetyl-L-carnitine was administered alone, Bax expression was not affected.

## Discussion

Ag-NPs are being widely used in FDA regulated products. According to the Nanotechnology Consumer Products Inventory there are 1628 consumer products that self-identify as containing nanomaterials, including 383 products that contain Ag-NPs, such as baby blankets and children's plush toys. Ag-NPs have been used for antibacterial purposes and for water purification (Sharma and Sharma, 2007). Therefore, frequent and long-term exposure to Ag-NPs is not a rare phenomenon. Ag-NPs may enter the humans orally, dermally, or by inhalation. Once Ag-NPs enter the blood stream they are distributed throughout the body including the central nervous system (CNS). Although the CNS is protected by the BBB from many compounds, both endogenous and exogenous, Sharma and Sharma (2007) reported that the function of the BBB was compromised in rats treated with Ag-NPs. These investigators showed that the daily intraperitoneal injection of 50 mg/kg of Ag-NPs for 7 consecutive days caused minor changes in behaviors such as decreased inclined plane angle and stride length. Recently, it was demonstrated that inhaled nanoparticles can translocate directly to the olfactory bulb in brain (Elder et al., 2006). Moreover, compared with other nanoparticles such as copper or aluminum, Ag-NPs seem to be more potent in causing breakdown of the BBB, as indicated by radioiodine tracer (Sharma and Sharma, 2007). At the cellular level, Ag-NPs have been shown to change neuronal cell morphology, reduced myelin, and induce gliosis (Sharma and Sharma, 2007). Recently, Zhang et al. (2013) injected Ag-NPs into the tail veins of rats at 45 mg/kg/day for 3 days and observed reduced locomotor activity and decreased rearing frequency. These behavioral alterations noted after Ag-NP exposures suggest effects on neural tissues and/or networks and merit further investigation.

There seems to be consensus that nanoparticle-induced neurotoxicity does occur, particularly in settings in which the brain is most vulnerable such as during developmental or in the presence of pathological processes or genomic predisposition. The causality of nanoparticle-induced cytotoxicity is presently unclear and probably multifactorial, although size, shape, coating material and experimental conditions all likely contribute to cytotoxicity (Wang et al., 2008; Xia et al., 2008; Ray et al., 2009; Lu et al., 2010). Meanwhile, due to the complexity (incomplete anatomical structure, e.g., BBB and its immature functions) and temporal features that can come into play in the expression of developmental neurotoxicity, infants and/or young children can be more vulnerable to toxicants.

Since there is no practical way to obtain relevant dose-response and time-course data on nanoparticle-induced neural damage in infants and children, it is imperative that these be obtained from appropriate laboratory models. To date, a number of such studies have been carried out in rodents, but there have been questions as to whether the toxic effects of treatments observed in animals or in tissue derived from animals adequately mirror human responses. To address this issue, NSCs from both rats and humans were used in the present study, which provided important comparative data. The data in Figure 2 show that the rat and human NSCs were similarly sensitive to toxic effects produced by exposure to Ag-NPs, namely increases in LDH release and decreases in mitochondrial viability. These data suggest that, at least in the case of Ag-NPs, both rat and human responses are very similar, and support the concept that the biological pathways affected by Ag-NPs might be conserved across species and are shared by rats and humans alike.

The data from the present study also indicate that various factors likely contribute to determining the responses of NSCs to external stimuli. NSC-based models seemingly provide opportunities to recapitulate critical aspects of CNS development *in vitro* and should, therefore, provide opportunities to study those specific biological factors critical for normal development. Here, the data show that 24 h exposures to Ag-NPs damaged both rat and human NSCs in a dose-dependent manner. The pediatric population, particularly during sensitive developmental stages characterized by NSC proliferation, differentiation and migration, is very likely to be more vulnerable to the adverse effects of a variety of neuroactive insults, including exposure to Ag-NPs.

Ag-NP exposures caused increases in Bax protein expression and the number of TUNEL–positive cells, suggesting increased apoptosis. The role of the Bax in apoptosis has been intensively studied. As previously reported, Bax may open pores on the outer mitochondrial membrane, allowing exit of cytochrome C (Green and Reed, 1998). In addition to apoptosis, the increased LDH release indicated cell membrane corruption, which is usually associated with necrosis.

Several studies have reported that Ag-NPs affect mitochondrial function and the antioxidant defense system (Braydich-Stolle et al., 2005; Hussain et al., 2005, 2006). Since silver ions react readily with thiol groups including glutathione and those located at the mitochondrial inner membrane, the adverse effects of Ag-NPs may not be exclusively attributed to them, but may involve the silver ions that most assuredly exist in some equilibrium them. L-carnitine is also well known to preserve cellular membrane integrity and play a critical role in mitochondrial oxidation of long-chain fatty acids (Loots et al., 2004). Thus, elevated levels of L-carnitine should, at least theoretically, enhance the capability of the mitochondrial antioxidant system and decrease the incidence of free radical-induced lipid peroxidation (Yildiz et al., 1996). In the present study, the fact that co-administration of acetyl-L-carnitine blocked Ag-NP-induced NSC death further indicates that this toxic effect is closely related to mitochondrial membrane integrity and function and that ROS likely play an important role.

The generation of ROS is a predominant pathway leading to cytotoxicity (Xia et al., 2008). Evidence for the role of oxidative stress in nanoparticle-induced neurotoxicity has been generated in studies utilizing agents that diminish or block oxidative stress. In the current study, elevated levels of 8-oxo-dG released in culture medium after Ag-NP exposure suggest enhanced oxidative damage due to an increased generation of ROS. Meanwhile, the protective effects of acetyl-L-carnitine also suggest a role for ROS in Ag-NP-induced cell damage. Therefore, direct measurement of ROS becomes important for confirming the involvement of ROS in Ag-NP-induced NSC damage. In the present study, mitochondrial ROS production was evaluated using a DCF assay, which utilizes a fluorescent probe specific for mitochondrial ROS. The data demonstrate that 24 h exposures of NSCs to Ag-NPs resulted in a significant increase in mitochondrial ROS production suggesting, consistent with previous reports (Fan and Lu, 2005; Xia et al., 2008), that nanoparticle-induced cytotoxicity is largely mediated by the induction of ROS.

Given the importance of NSC proliferation in neurogenesis and normal development we also sought to determine whether NSC damage seen after Ag-NP exposure could be associated with interruptions in cell division cycles. Here, NSC proliferation was examined using a commercially available EdU incorporation method. The data show that Ag-NPs can initiate oxidative mitochondrial damage and dysfunction and subsequently affect NSC proliferation by slowing down or even stopping cell division, effects that contribute to NSC damage.

In summary, our findings suggest that Ag-NP-induced cell death in rat and human NSC models seem to be both apoptotic and necrotic in nature, and that it occurs in a dose and exposure duration dependent manner. This study provides direct evidence that elevated mitochondrial ROS plays a critical role in Ag-NP-induced neurodegeneration. Co-administration of the anti-oxidant agent acetyl-L-carnitine significantly attenuates the adverse effects of Ag-NPs. In addition, Ag-NPs can directly affect NSC proliferation.

### Disclaimer

This document has been reviewed in accordance with United States Food and Drug Administration (FDA) policy and approved for publication. Approval does not signify that the contents necessarily reflect the position or opinions of the FDA. The findings and conclusions in this report are those of the authors and do not necessarily represent the views of the FDA.

### Conflict of interest statement

The authors declare that the research was conducted in the absence of any commercial or financial relationships that could be construed as a potential conflict of interest.
